# Theoretical Study on All-Dielectric Elliptic Cross Metasurface Sensor Governed by Bound States in the Continuum

**DOI:** 10.3390/ma16052113

**Published:** 2023-03-06

**Authors:** Haocheng Cai, Xiaoxu Yu, Luhong Mao

**Affiliations:** 1 School of Electrical and Information Engineering, Tianjin University, Tianjin 300072, China; 2 Tianjin Navigation Instrument Research Institute, Tianjin 300131, China

**Keywords:** metasurface, sensor, bound states in the continuum, information encoding

## Abstract

The appearance of all-dielectric micro–nano photonic devices constructed from high refractive index dielectric materials offers a low-loss platform for the manipulation of electromagnetic waves. The manipulation of electromagnetic waves by all-dielectric metasurfaces reveals unprecedented potential, such as focusing electromagnetic waves and generating structured light. Recent advances in dielectric metasurfaces are associated with bound states in the continuum, which can be described as non-radiative eigen modes above the light cone supported by metasurfaces. Here, we propose an all-dielectric metasurface composed of elliptic cross pillars arranged periodically and verify that the displacement distance of a single elliptic pillar can control the strength of the light–matter interaction. Specifically, when the elliptic cross pillar is C_4_ symmetric, the quality factor of the metasurface at the Γ point is infinite, also called the bound states in the continuum. Once the C_4_ symmetry is broken by moving a single elliptic pillar, the corresponding metasurface engenders mode leakage; however, the large quality factor still exists, which is called the quasi-bound states in the continuum. Then, it is verified by simulation that the designed metasurface is sensitive to the refractive index change of the surrounding medium, indicating that it can be applied for refractive index sensing. Moreover, combined with the specific frequency and the refractive index variation of the medium around the metasurface, the information encryption transmission can be realized effectively. Therefore, we envisage that the designed all-dielectric elliptic cross metasurface can promote the development of miniaturized photon sensors and information encoders due to its sensitivity.

## 1. Introduction

The development of metamaterial photonics has been greatly promoted since Pendry et al. proposed a composite structure composed of high refractive index dielectric cylinders and verified that the structure has effective negative permeability [1]. The typical metamaterial is the metal split-ring resonator operating in the microwave band. However, due to the inherent ohmic loss of metals, potential applications of metal metamaterials are hindered [2,3]. Excitingly, the proposal of all-dielectric metasurfaces based on high refractive index dielectric materials provides an excellent platform for the exploration of lowloss subwavelength photonic devices [4]. In recent years, all-dielectric metasurfaces have been widely harnessed in many fields, such as beam focusing [5,6,7,8,9], holography [10,11,12], imaging [13,14,15], structured light generators [16,17,18,19], etc.

The function of restricting electromagnetic waves possessed by numerous metasurfaces can be attributed to strong resonance, whose corresponding quality factor (Q factor) is an important indicator to measure the strength of light–matter interaction. Generally, tailoring the shape, size and periodicity of all dielectric meta-atoms can increase the Q factor and enhance the local electromagnetic field, which can be employed in a wide range of realms, such as sensing [20,21,22], optical switch [23], and high-order harmonic generation [24]. Recently, the proposal of metasurfaces supporting bound states in the continuum (BICs) provides a new scheme for further enhancing the light–matter interaction. Specifically, under the incidence of electromagnetic waves, some specific modes generated by metasurfaces above the light cone carry infinite Q factors, which cannot be excited by radiation from the far field [25,26]. However, for practical optical devices, due to the influence of material absorption and fabricating technology, they have finite Q factors and narrow resonance linewidths, called Quasi BICs. The general strategy to achieve Quasi BICs is to break the structural symmetry of the resonant unit [27,28,29,30]. In other words, rotating the resonant unit in a specific modality or adding (reducing) a part of the initial resonant unit can generate Quasi BICs with sharp Fano lines under the incidence of electromagnetic waves [31,32,33,34]. For example, Sinclair et al. proposed that the removal of highly symmetrical resonant units can induce coupling between orthogonal modes, thereby generating Fano resonance with a high-quality Q factor. Furthermore, they verified the feasibility of the scheme through experiments [31]. Tittl et al. broke the C_2_ symmetry of the resonant unit by changing the tilt angle of a pair of elliptic pillars, and produced Quasi BICs which can be exploited for highly sensitive molecular sensors [33]. In addition, by using a metasurface that supports a symmetry-protected Quasi BIC, the near-field imaging can be realized [34].

Here, we propose an all-dielectric metasurface consisting of elliptic cross silicon pillars with C_4_ symmetry arranged periodically. By calculating the eigenmodes and transmission spectra excited by different modes of electromagnetic waves in C_4_ symmetry, it is verified that the metasurface has an infinite Q factor at the Γ point, that is, the metasurface supports BIC. After introducing an asymmetric parameter to break C_4_ symmetry, the metasurface can generate sharp Fano resonance under the incidence of electromagnetic waves. The relationship between the radiative Q factor and the asymmetric parameters manifests that the BICs supported by the metasurface are symmetry protected BICs. Once different refractive index mediums are introduced into the gap of the elliptic cross silicon pillars, the metasurface exhibits a sensitivity to the change of the refractive index. Moreover, based on the high Q properties of Quasi BICs, information encoding can be realized effectively by selecting specific meta-atoms to construct a metasurface. By changing the refractive index of the medium around the metasurface, information encryption of near-field imaging is realized, which effectively verifies the feasibility of information encoding. These above schemes provide a new working platform for real-time liquid refractive index detection and information encoding.

## 2. Results and Discussion

**Figure 1:**
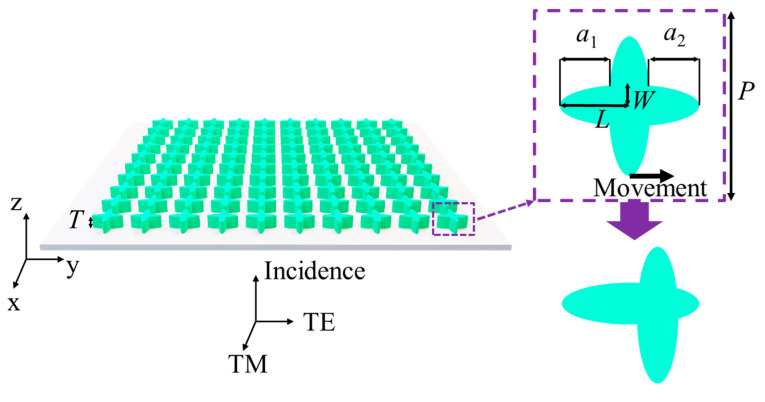
Schematic diagram of all-dielectric metasurface supporting BICs. The metasurface is composed of an elliptic cross silicon pillar, with a thickness of *T* = 150 μm. The substrate is made of silica, and the corresponding thickness is *h* = 50 μm. The illustration is a top view of a single atomic element. The period is *p* = 300 μm, and the corresponding major and minor semi-axes of a single elliptic pillar are L = 120 μm and W = 30 μm, respectively.

Here, in order to show the excitation effect of symmetry on BICs supported by the metasurface, we propose an all-silicon elliptic cross metasurface (refractive index *n*_2_ = 3.45) placed on a substrate composed of silica (refractive index *n*_1_ = 1.45), whose corresponding schematic diagram is illustrated in Figure 1. It can be seen from Figure 1 that the thickness of the metasurface is *T* = 150 μm, and the thickness of the corresponding substrate is *h* = 50 μm. The period of the meta-atom is *p* = 300 μm, and the corresponding major and minor semi-axes of a single elliptic pillar are *L* = 120 μm and *W* = 30 μm, respectively. When the values of *a*_1_ and *a*_2_ are equal, the meta-atom is C_4_ symmetric. Moving a single elliptic pillar makes *a*_1_ ≠ *a*_2_, which can break the C_4_ symmetry of the meta-atom. Next, we will analyze the BICs supported by the designed metasurface under the incidence of TM and TE modes, respectively.

**Figure 2:**
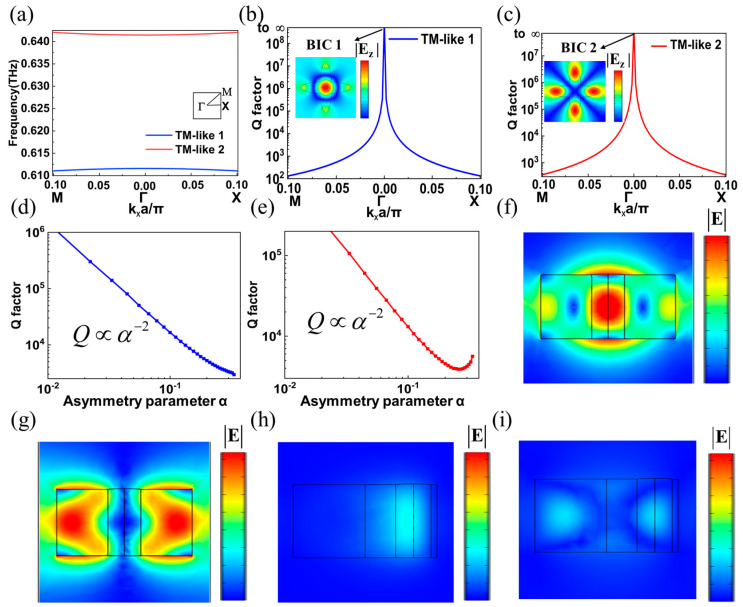
(**a**) Band diagram of TM-like mode in an elliptic cross pillar with C_4_ symmetry. The illustration shows a k-space diagram, and Γ point shows a highly symmetric point. (**b**,**c**) The radiation Q factor of TM-like 1 and TM-like 2 modes in an elliptic cross pillar with C_4_ symmetry, and the illustration shows the electric field distribution at Γ point. (**d**,**e**) The correlation curve of radiation Q factor of TM-like 1 (TM-like 2) mode and asymmetric parameter *α*. (**f**,**g**) When the asymmetric parameters meet the condition: *α* = 0, the electric field distributions of TM-like 1 and TM-like 2 modes at xoz plane. (**h**,**i**) When the asymmetric parameters meet the condition: *α* = 0.8, the electric field distributions of TM-like 1 and TM-like 2 modes at xoz plane.

It is worth emphasizing that the BICs supported by a metasurface have a strong interaction with infinite Q factors and zero leakage. Electromagnetic waves of a particular frequency coexist with extended states in the radiative continuum, while remaining perfectly localized in the absence of radiation. Therefore, BICs with radiation leakage can be obtained by adjusting some geometric or structural parameters of the metasurface. One of the more common ones is to break the symmetry of the structure and obtain the symmetry-protected quasi-BICs. Thus, in order to verify that the designed metasurface can support symmetry-protected BICs, COMSOL Multiphysics software is harnessed to build a single meta-atom model and calculate the eigenmodes (see Methods). Figure 2a shows two band diagrams of the TM-like modes in the case of C_4_ symmetry. Among them, the radiation Q factors of TM-like 1 and TM-like 2 modes are illustrated in Figure 2b,c, respectively. The frequency eigenvalue of quasi-BIC solved in COMSOL software (COMSOL Multiphysics 5.6, Burlington, MA, USA) is a complex number, expressed as *ω*-i*γ*, with the real part representing the frequency center and the imaginary part representing the radiation rate. Therefore, the Q value can be obtained based on the above results. Here, the equation for calculating Q factor is: Q = *ω*/2*γ*, where *ω* represents the resonant frequency and *γ* represents the corresponding radiation rates. Apparently, these two modes have an infinite Q factor at Γ point. The insets show the electric field distribution of the corresponding |**E**_z_|: when the frequencies are 0.611 and 0.641 THz, the electric field at the Γ point is confined to the elliptic cross pillar. The electric field of TM-like 1 mode is concentrated at the center of the elliptic cross pillar, while the electric field of TM-like 2 mode is mainly distributed at the boundary. Then, the C_4_ symmetry is broken by moving a single elliptic pillar of the elliptic cross pillar along the x-direction. Here, the asymmetric parameter is defined as: *α* = |(*a*_1_ − *a*_2_)/(*a*_1_ + *a*_2_)|. Furthermore, the movement distance of a single elliptic pillar along the x-direction is: *d* ≈ |*a*_1_ − *a*_2_|/2. As shown in Figure 2d, there is an inverse relationship between the radiation Q factor of TM-like 1 mode and the square of the asymmetric parameter *α*, which is consistent with reference [32]. Figure 2e shows the radiation Q factor of TM-like 2 mode corresponding to different asymmetric parameters. It can be concluded that the radiation Q factor of such mode is basically inversely proportional to the square of the asymmetric parameter *α*. Figure 2f,h shows the electric field distribution of the TM-like 1 mode in the xoz plane with the asymmetric parameters *α* = 0 and *α* = 0.8. Of particular emphasis, the absolute value of electric field is considered here. It can be seen that in the case of C_4_ symmetry, TM-like 1 mode is bound inside the entire elliptic cross pillar, and after breaking the C_4_ symmetry, TM-like 1 mode will leak into the far field. However, part of the electric field is still distributed inside the elliptic cross pillar, and such a mode is called a Quasi BIC 1. Similarly, we obtained the electric field distribution of TM-like 2 mode in xoz plane with asymmetric parameters *α* = 0 and *α* = 0.8 through simulation, as shown in Figure 2g,i. The results exhibit that the TM-like 2 mode is confined inside the dielectric pillar with the asymmetric parameter *α* = 0, while the strong leakage occurs with the asymmetric parameter *α* = 0.8. The above results demonstrate that the TM-like 1 and TM-like 2 modes supported by the designed metasurface in the case of C_4_ symmetry are BIC 1 and BIC 2 at Γ points, respectively, and these two BICs belong to the symmetry-protected BICs.

**Figure 3:**
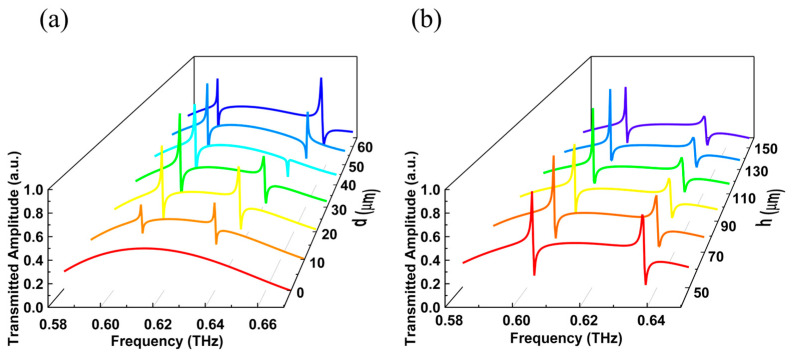
(**a**) When the movement distances of a single elliptic pillar are *d* = 0 μm, 10 μm, 20 μm, 30 μm, 40 μm, 50 μm and 60 μm, the transmission amplitude under the incidence of TM mode. The thickness of the substrate is *h* = 50 μm. (**b**) When the thicknesses of the substrate are *h* = 50 μm, 70 μm, 90 μm, 110 μm, 130 μm and 150 μm, the transmission amplitude under the incidence of TM mode. The movement distance of the single elliptic pillar is *d* = 20 μm.

Next, CST Studio Suite software (CST STUDIO SUITE 2019, Computer Simulation Technology AG, Darmstadt, Germany) is used to build a single elliptic cross pillar (see Methods). We set TM mode input at the incident port and TM mode output at the outgoing port. The transmitted amplitude of TM mode at different frequencies in Figure 3a is obtained by employing a frequency domain solver. Here, the thickness of the substrate is maintained at 50 μm. It is worth noting that when the movement distance *d* of a single elliptic pillar is not zero, Fano resonances will occur around 0.61 and 0.64 THz, respectively. When *d* increases from 10 μm to 60 μm, the lower resonant frequency decreases by 4.9 GHz, which means that the increase in *d* will lead to a blue shift in the resonant frequency. The linewidth of the Fano resonance decreases with the decrease in *d*, and disappears in the case of *d* = 0 μm, again confirming the presence of BIC 1 and BIC 2 in Figure 2b,c. In addition, when the movement distance of a single elliptical pillar is *d* = 20 μm, the amplitude changes of Fano resonances generated near 0.61 and 0.64 THz is more intense, which may be applied to narrowband bandpass filtering. With this, the transmitted amplitude of the TM mode with different thicknesses of substrate *h* is further studied. Here, the control variate methods are harnessed to maintain the movement distance of the elliptic pillar as *d* = 20 μm. The transmitted amplitude of TM mode is calculated when the thicknesses of substrate are *h* = 50 μm, 70 μm, 90 μm, 110 μm, 130 μm and 150 μm, as shown in Figure 3b. It can be seen from Figure 3b that with the increase in the thickness of the substrate *h*, the Fano resonance has a redshift. Meanwhile, with the change of thickness of the substrate *h*, the maximum amplitude variation in Fano resonance near 0.61 THz is inapparent, while the maximum amplitude variation in Fano resonance near 0.64 THz is more obvious. Therefore, the change of thickness of the substrate *h* will cause the frequency shift of the transmitted spectrum of TM mode.

**Figure 4:**
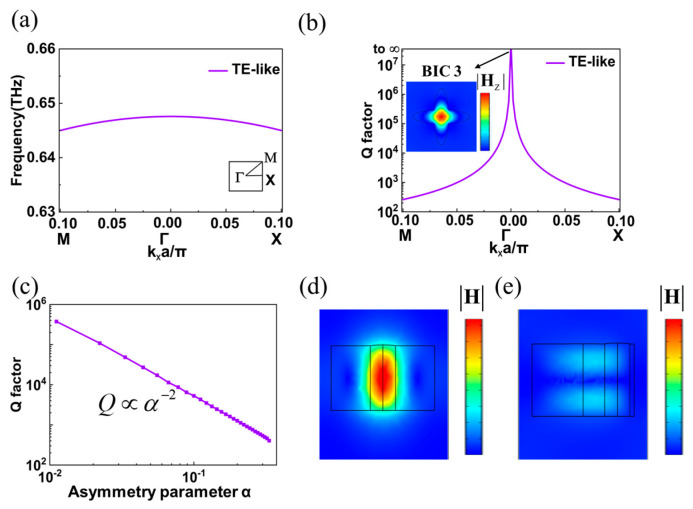
(**a**) Band diagram of transverse electric-like (TE-like) mode in an elliptic cross pillar with C_4_ symmetry. (**b**) The radiation Q factor of TE-like mode in an elliptic cross pillar with C_4_ symmetry, and the illustration shows the electric field distribution at Γ point. (**c**) The correlation curve of radiation Q factor of TE-like mode and asymmetric parameter *α*. (**d**) When the asymmetric parameters meet the condition: *α* = 0, the magnetic field distribution of TE-like mode. (**e**) When the asymmetric parameters meet the condition: *α* = 0.8, the magnetic field distribution of TE-like mode.

Two cases of TM-like mode in elliptic cross pillar are discussed and analyzed above. Next, we will further analyze the TE-like mode. Similar to the method adopted in Figure 2a, the band diagram of TE-like mode under C_4_ symmetry is obtained through COMSOL Multiphysics software (see Methods, COMSOL Multiphysics 5.6, Burlington, MA, USA), as shown in Figure 4a. Figure 4b shows the corresponding radiation Q factor, while the illustration is the magnetic field distribution (|**H**_z_|) at Γ point (BIC 3). It can be seen from the illustration that when the frequency is 0.646 THz, the magnetic field |**H**_z_| is mainly concentrated in the center of the elliptic cross pillar. Figure 4c shows the relationship curve between the radiation Q factor and the asymmetric parameter *α* after moving a single elliptic pillar: similar to the results in Figure 2c, the radiation Q factor is basically inversely proportional to the square of the asymmetric parameter *α*. In addition, Figure 4d,e show the |**H**| distributions of the TE-like mode on the xoz plane with the asymmetry parameter *α* = 0 and *α* = 0.8, respectively. Clearly, the magnetic field is bound in the dielectric pillar with the asymmetry parameter *α* = 0, while strong magnetic field leakage occurred with the asymmetry parameter *α* = 0.8. In short, BIC 3 at Γ point (0.646 THz) in Figure 4b is a symmetry-protected BIC.

**Figure 5:**
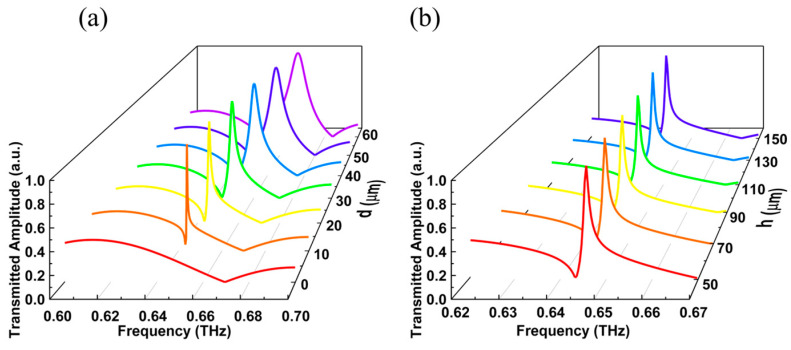
(**a**) When the movement distances of a single elliptic pillar are *d* = 0 μm, 10 μm, 20 μm, 30 μm, 40 μm, 50 μm and 60 μm, the transmission amplitude under the incidence of TE mode. The thickness of the substrate is *h* = 50 μm. (**b**) When the thicknesses of the substrate are *h* = 50 μm, 70 μm, 90 μm, 110 μm, 130 μm and 150 μm, the transmission amplitude under the incidence of TE mode. The movement distance of the single elliptic pillar is *d* = 20 μm.

Further, a single elliptic cross pillar is constructed using CST Studio Suite software with the frequency range of 0.6–0.7 THz. We set TE mode input at the incident port and TE mode output at the outgoing port. The transmitted amplitude curve of TE mode in Figure 5a is obtained by employing the frequency domain solver. Here, the thickness of the base is maintained as *h* = 50 μm. Once the movement distance *d* of the elliptic pillar is increased, the linewidth of Fano resonance gradually increases, and the transmitted spectrum has a slight blue shift. Apparently, when *d* = 0 μm, the linewidth of Fano resonance in the transmitted spectrum disappears, which verifies that the designed elliptic cross pillar can support BIC 3 under C_4_ symmetry. In addition, in the case of *d* ≠ 0 μm, the Fano resonance in the transmitted spectrum is called Quasi BIC 3. The movement distance *d* of the elliptic pillar is set as 20 μm, and the transmitted amplitude of TE mode with different thickness *h* has been calculated (see Figure 5b). Figure 5b manifests that with the increase in *h*, Fano resonance in transmitted amplitude curve of TE mode has a blue shift, while the corresponding linewidth has hardly changed. Therefore, it can be determined that the change of *h* mainly affects the frequency of Fano resonance, but hardly change the linewidth of Fano resonance.

**Figure 6:**
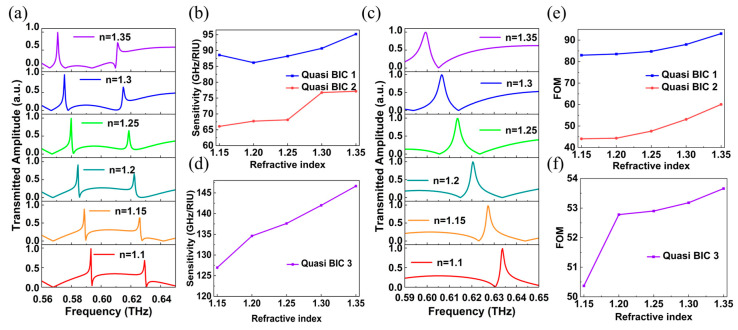
(**a**) The relation between the transmitted amplitude and frequency generated by the meta-atom under the incidence of TM mode. Here, *n* represents the refractive index of the material in the gap between adjacent elliptic cross pillars. (**b**) The sensitivities of Quasi BIC 1 and 2 in TM-like mode is calculated by taking the refractive index difference between two adjacent refractive indexes as a unit. (**c**) The relation curve between transmitted amplitude and frequency generated by meta-atom under the illumination of TE mode. (**d**) Sensitivity of Quasi BIC 3 engendered by meta-atom under the incidence of TE mode. (**e**,**f**) Figure of merit (FOM) of the three Quasi BICs under the incidence of TM and TE modes.

To evaluate the sensing performance of the designed all-dielectric elliptic cross metasurface, the refractive index sensing capability of the metasurface is quantified. Concretely, a medium with different refractive index *n* is filled in the gap of the metasurface to simulate the situation of immersing the metasurface into a solution mixture with different refractive index. As shown in Figure 6a, the thickness of the substrate is set as *h* = 50 μm, and the movement distance of a single elliptic pillar is *d* = 20 μm, then the transmitted spectra at *n* = 1.1, 1.15, 1.2, 1.25, 1.3 and 1.35 are obtained through simulation. When the refractive index is *n* = 1.1, the transmission curve of the designed metasurface under the incidence of TM mode engenders Fano resonance at 0.593 THz (Quasi BIC 1) and 0.631 THz (Quasi BIC 2). In addition, with the increase in the refractive index *n*, the two Fano resonances in the transmitted curve have red shifted, and the linewidths of Quasi BIC 1 and Quasi BIC 2 exist slightly changed. To further measure the performance of the sensor, the sensitivity (S = |Δ*f*/Δ*n*|) is calculated according to the frequency offset of Quasi BIC 1 and Quasi BIC 2 in the transmitted curve with different refractive index *n* in Figure 6a, as plotted in Figure 6b. Among them, Δ*n* is the difference between adjacent refractive indices (in Figure 6a), and Δ*f* is the frequency shift of the resonant frequency in response to the refractive index change Δ*n*. The blue curve and the red curve represent the sensitivity of Quasi BIC 1 and Quasi BIC 2, respectively. It is not difficult to find that the sensitivity of Quasi BIC 1 is always higher than 85 GHz/RIU, and the sensitivity of Quasi BIC 2 is in the range of 65–77 GHz/RIU. Therefore, under the incidence TM mode incidence, the sensing performance of Quasi BIC 1 generated by the metasurface is better than that of Quasi BIC 2.

Furthermore, the simulated results of the metasurface filled with different media under the illumination of TE mode are obtained through CST Studio Suite software (see Figure 6c); with the increase in the refractive index *n* of the filled media, the resonant frequency at Quasi BIC 3 has a significant red shift, and the resonant linewidth increases accordingly. As the refractive index *n* increases from 1.1 to 1.35, the corresponding frequency of Quasi BIC 3 moves from 0.635 to 0.599 THz. Figure 6d shows the sensitivity after the change of refractive index *n*. According to Figure 6d, it can be determined that under the incidence of TE mode, when the difference between adjacent refractive indices remains the same, increasing the refractive index of the filled medium can yield greater sensitivity. In addition, the sensitivity of excited Quasi BIC 3 after the incidence of TE mode is always greater than 120 GHz/RIU, signifying that Quasi BIC 3 has better sensing performance than excited Quasi BIC 1 and 2 after the incidence of TM mode. Wang et al. ‘s work mentions that sensitivity on the order of 10^2^ GHz/RIU can be used for sensing [35], so it can be concluded that when the thickness of substrate is *h* = 50 μm and the movement distance of a single elliptic pillar is *d* = 20 μm, the constructed metasurface is sensitive to the refractive index of the surrounding medium, which may be suitable for sensing. It is worth emphasizing that the sensor with three peaks can improve the sensing accuracy compared with the sensor with only one peak under two incident modes. Apart from this, to facilitate the comparison of the sensing performance of sensors applied to different wavebands, Figure of Merit (FOM) is proposed, which is defined as [36]: FOM = S/FWHM, where FWHM is full width at half maxima of resonant peak. It is worth emphasizing that the greater the value of FOM, indicating that the sensor has better sensing performance. Thus, according to the above formula, the FOM of the three Quasi BICs in Figure 6b,d are calculated (see Figure 6e,f). It can be found that the FOM of Quasi BIC 1 is higher than that of the other two Quasi BICs, and the corresponding FOM reached a maximum of 93 RIU^−1^. Interestingly, with the increase in the refractive index *n*, the sensitivity and FOM of the three Quasi BICs all illustrated an increasing trend, which verified that the interaction between the medium around the metasurface and the electric field is enhanced with the increase in the refractive index *n*. Meanwhile, performance comparison between such sensor and some other recently developed sensors [37,38,39,40,41,42] is listed in Table 1. Comparatively, the sensing performance of the designed metasurface is not optimal, which may be attributed to the characteristics of the material itself [43].

**Figure 7:**
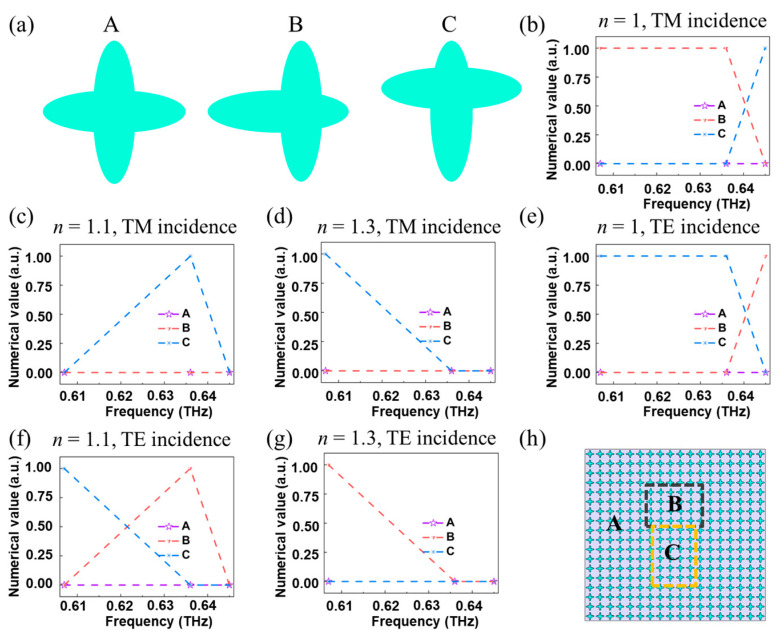
(**a**) The three representative meta-atoms harnessed to encode information are denoted as A, B and C. (**b**–**d**) When the refractive index *n* = 1, 1.1 and 1.3, the number of codes of the meta-atom at different frequencies under TM incidence. (**e**–**g**) When the refractive index *n* = 1, 1.1 and 1.3, the number of codes of the meta-atom at different frequencies under TE incidence. (**h**) The structure diagram of the constructed information encoder.

The above results have confirmed that the resonance in the transmission spectrum of the designed element at TM (TE) incidence shifts with the change of refractive index of the medium around the element atoms. Here, we take advantage of this property to encode the information based on near-field imaging [34]. As shown in Figure 7a, three representative meta-atoms are carefully selected, denoted as A, B and C respectively. Thereinto, A is the unit with C_4_ symmetry, B is the unit formed after moving a single elliptic pillar by 20 μm, and C is the unit formed after rotating B by 90° counterclockwise. For efficient numerical coding, the transmission amplitudes below 0.45 are defined as 0, and the transmission amplitudes above 0.45 are defined as 1. Figure 7b plots the numerical codes of these three meta-atoms at frequencies of 0.607, 0.636 and 0.645 THz under TM incidence when the refractive index of the surrounding medium is *n* = 1. Meanwhile, Figure 7c,d display the numerical results when the refractive index of surrounding medium is 1.1 and 1.3 respectively. It can be seen that as *n* changes, the numerical information of these meta-atoms at the same frequency changes. Furthermore, Figure 7e–g shows the results of the selected atoms under TE incidence when the refractive index of the surrounding medium is 1, 1.1 and 1.3. With these, we construct a metasurface to show the function of information coding (see Figure 7h), which is composed of 18 × 18 units. It can be seen that the area in the black box is composed of meta-atom B, the area in the yellow box is composed of meta-atom C, and the other areas are composed of meta-atom A.

**Figure 8:**
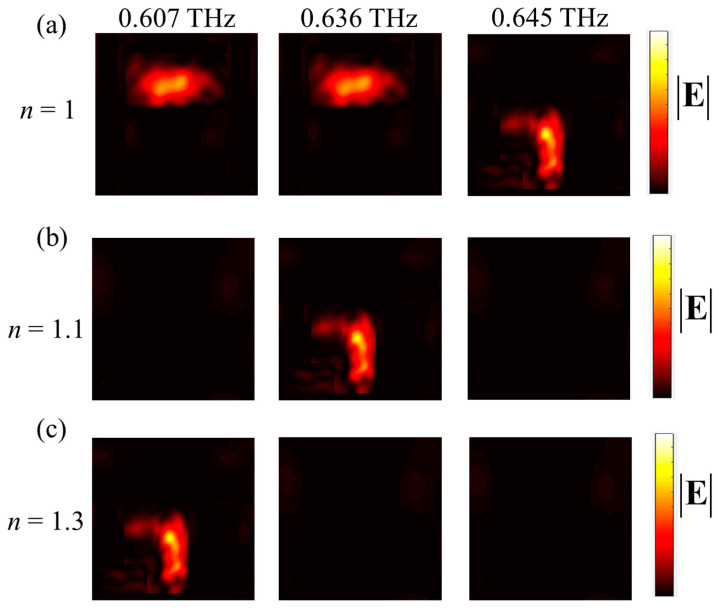
(**a**–**c**) The near-field intensity distributions of the designed information encoder at TM incidence, when the refractive index of the surrounding medium is 1, 1.1 and 1.3.

Then, the near-field intensity distribution of the metasurface designed in the air under the normal incidence of TM wave is obtained by simulation, as shown in Figure 8a (where TM incidence is considered as an example). It is obvious that the designed metasurface has the same intensity distribution when the frequencies are 0.607 and 0.636 THz: some regions with stronger energy form a pattern of ‘-’, while others have weaker energy. In addition, when the frequency is 0.645 THz, some regions with stronger energy form a pattern of ‘**|**’. Figure 8b depicts the intensity distribution at three frequencies under TM incidence when *n* = 1.1: when the frequency is 0.636 THz, the corresponding intensity distribution carries obvious patterns, while no obvious patterns are found at the frequencies of 0.607 and 0.645 THz. Similarly, when the refractive index of the surrounding medium is adjusted to 1.3, the designed metasurface has different intensity distributions at the above three frequencies. It can be found that when the frequency is 0.607 THz, the intensity distribution generated has a ‘|’ pattern, while the intensity distribution of the other two frequencies has no obvious pattern. In conclusion, the metasurface constructed based on three meta-atoms can generate specific near-field images under TM and TE incidences. Meanwhile, by changing the refractive index of the medium around the metasurface, different intensity distributions can be generated at the same frequency, which may be used for information encryption.

It is common knowledge that ordinary resonance generally has a wide working frequency band, which will cause that even if the central frequency is unknown during decryption, the result close to the real information may be obtained in a larger bandwidth near the central frequency. The advantages of a Quasi-BIC mode are high Q factor and narrow working frequency band, so it is necessary to know the monitoring frequency accurately in advance for decryption, which enhances the difficulty of decryption. With this, we proposed two simple application conceptions for the mentioned information encryption. First, encoding information encryption: by defining ‘-’ as 2, ‘|’ as 1, and none as 0, a ternary cipher compilation system can be obtained. This system can encrypt information through frequency, polarization direction and refractive index. For example, defines a code 3611 TM, it corresponds to 0.636 TH_Z_, 1.1 refractive index and TM polarization (such information can be designed to be more secretive). Then the 3611 TM encrypted information can be converted to the near-field field strength of ‘|’, that is, 1. Suppose that a letter A needs to be transmitted, and the binary code of A is ‘01000001′, which is converted to hexadecimal ‘2102′. For a unique 16 × 16 lattice, it can be compiled through 3610 TM, 3611 TM, 4513 TM, 0710 TM (where TM incidence is considered as an example). The advantage of this design is that the same information can be obtained in many ways to enhance deciphering difficulty. Second is the graphics encryption: by placing independent regions similar to the B and C regions at different angles, a basic graph can be constructed with ‘-’, and the near-field graph can only be decrypted with specific polarization, frequency and refractive index values, while the wrong polarization, frequency and refractive index information cannot obtain the correct graph. Hence, such above coding encryption scheme can be employed as a supplement to hardware encryption equipment and software encryption, improving the confidentiality of data.

## 3. Conclusions

In conclusion, we propose and verify that the all-dielectric metasurface constructed by elliptic cross pillars with C_4_ symmetry can support three different symmetry-protected BICs. Under the excitation of the TM mode, two Fano resonances can appear by adjusting the movement distance of a single elliptic pillar, and the linewidths of these two resonances gradually decrease and disappear with the decrease in movement distance, accompanied by a slight frequency shift. The change in thickness of the substrate mainly affects the shift of resonance frequency. Similarly, under the excitation of the TE mode, the change of movement distance mainly changes the resonant linewidth of Quasi BIC 3, while the thickness of the substrate mainly affects the corresponding frequency of Quasi BIC 3. After introducing different refractive index media between adjacent elliptic cross pillars, the transmitted spectrum generated by the metasurface exists as a significant shift. Sensitivity is introduced to quantify the sensitive ability of the metasurface to the change of the refractive index. The simulated results show that the sensitivity of the designed metasurface under the incidence of the TE mode is greater than 120 GHz/RIU, indicating that this design scheme may promote the development of real-time liquid refractive index detection and biosensors. In addition, information can be encoded effectively with high encryption by combining the specific frequency with the refractive index of the medium around the metasurface.

**Methods:** Eigenmode solution: After building the model, periodic boundary conditions are applied along the x- and y-directions. Such a model is symmetrical up and down, so we can have two perfect sets of energy bands: TE-like and TM-like. TE-like and TM-like are derived from TE and TM modes. Because the designed meta-atom does not have continuous translational symmetry in the z-direction, there is no real TE or TM mode, but it can be based on the symmetry plane of the meta-atom in the z-direction Γ. The direction of the point electric field and magnetic field defines the entire energy band as TE-like or TM-like, but the above premise is that the entire atomic element is mirror symmetric in the z-direction. If the meta-atom is located in the z-direction without mirror symmetry, such as air on one side and substrate on the other, the energy band obtained is neither perfectly TE-like or TM-like. Here, the model we choose is with the atoms as all air, so it has perfect two sets of energy bands. This requires us to add a perfect magnetic conductor (PMC) or perfect electric conductor (PEC) on the high symmetry plane, and at the same time, we can simplify the model—only need to calculate half of the region. The boundary condition set on its lower surface is PMC (TE-like) or PEC (TM-like). It should be noted that the thickness of the designed atomic element should be reduced by half. Then, different eigen modes and corresponding Q factors can be calculated by the eigen mode solver.

Spectrum solution: In CST Studio Suite software, the x-direction and y-direction are set as unit cell, the z-direction is set as open boundary, and the frequency range is set as 0.56–0.67 THz.

## Figures and Tables

**Table 1:** Comparison of sensor performance in recent years.

Reference	Freguency (THz)	Material	S (GHz/RIU)	FOM
[37]	0.5–1.1	Silicon	77	11.1
[38]	0.2–1.2	Gold	105	7.51
[39]	0.15–0.9	Aluminium	139.2	-
[40]	0.4–1.6	Graphene	177.7	59.3
[41]	555–568	Silicon nitride	-	163.5
[42]	0.2–2.1	LiTaO_3_	438	515
This work	0.5–0.7	Silicon	147	93

## Data Availability

Data underlying the results presented in this Letter are not publicly available at this time but may be obtained from the authors upon reasonable request.
